# Sarcoidosis in a young child with Alagille syndrome: a case report

**DOI:** 10.1186/1546-0096-10-32

**Published:** 2012-08-31

**Authors:** Melissa Mannion, Mem Zolak, David R Kelly, Timothy Beukelman, Randy Q Cron

**Affiliations:** 1 Department of Pediatrics, Division of Rheumatology, University of Alabama at Birmingham, Birmingham, AL, USA; 2 Department of Pediatrics, Division of Neonatology, University of Alabama at Birmingham, Birmingham, AL, USA; 3 Department of Pathology, University of Alabama at Birmingham, Birmingham, AL, USA

**Keywords:** Sarcoidosis, Alagille syndrome, Granulomatous angiitis, Granuloma, *JAG1*, *NOTCH1*, T lymphocyte

## Abstract

We report a now 6-year-old African-American male with both Alagille syndrome and pediatric sarcoidosis. With a prior *JAG1* mutation positive diagnosis of Alagille syndrome, he presented to the hospital with a subacute, predominantly respiratory febrile condition, eventually diagnosed as sarcoidosis. A liver biopsy revealed paucity of bile ducts and scattered epithelioid granulomas, while a skin biopsy showed granulomatous angiitis, a manifestation of sarcoidosis not yet reported in a pediatric patient. He has subsequently been treated with corticosteroids, mycophenolate mofetil, and infliximab with clinical response. Alagille syndrome and sarcoidosis have not yet been reported in the medical literature in the same patient to the best of our knowledge. We briefly review these two seemingly unrelated conditions and propose a possible common pathogenic mechanism.

## Background

Sarcoidosis is a systemic granulomatous disease of unknown etiology that may affect many organ systems. The lungs are the most commonly involved organs in adults. Adults with sarcoidosis often have lymphadenopathy and systemic manifestations, such as fever and weight loss. Diagnosis of pediatric sarcoidosis, as in adults, requires demonstration of non-caseating granulomata in one or more organs in the setting of consistent clinical or radiographic findings and the exclusion of infectious granulomatous conditions. There is no singular diagnostic lab test [1]. Derangement in calcium occurs in 30% of children with sarcoidosis and manifests as hypercalciuria, with or without hypercalcemia [2]. Hypercalcemia is more prevalent in affected children than adults. An elevated angiotensin converting enzyme (ACE) level can be found in sarcoidosis patients, however it is a less sensitive test in children than in adults and is thought to be a product of the epithelioid cells of the sarcoid granuloma [1]. In addition, serum lysozyme can be used as a marker for sarcoidosis. In one Japanese study, lysozyme had a sensitivity of 79% for predicting sarcoidosis. That study found that lysozyme levels tended to increase with the number of organ systems involved and the disease activity [3].

Pediatric sarcoidosis is rare, and its true incidence is unknown. A recent Danish study estimates the incidence at 0.22-0.27 per 100,000 children younger than 15 years of age but, different geographic regions or racial groups may have a higher prevalence [2]. Most cases of pediatric sarcoidosis are diagnosed in the pre-adolescent or adolescent age groups. By contrast, there is an early-onset form that occurs in children up to age 4 or 5 years and is usually characterized by boggy arthritis, rash, and uveitis [4]. Onset frequently occurs in the first year of life, and the classic presentation is rash (usually papular and erythematous) or arthritis (usually a boggy tenosynovitis) without pulmonary involvement, followed by uveitis. Although pulmonary involvement is not typical at diagnosis, it can develop later in the disease course. Blau syndrome is a triad of synovitis, uveitis and rash with an autosomal dominant inheritance of a mutation in CARD15 (caspase recruitment domain)/NOD2 (nucleotide binding oligomerization domain) on chromosome 16 [2,5] and may represent a substantial subset of what is called early-onset sarcoidosis. In addition to the familial Blau syndrome, there have been reports of sporadic mutations in CARD15 resulting in early onset sarcoidosis [6].

In contrast, older children and adolescents with sarcoidosis are often more similar to adults with lung involvement and constitutional symptoms [1,2]. Almost half of children with sarcoidosis show restrictive lung disease on pulmonary function testing [2]. If there is pulmonary involvement, hilar adenopathy is a hallmark finding on chest radiograph [1,2]. Peripheral lymphadenopathy is also a common finding in older children with sarcoidosis, such that peripheral lymph node enlargement occurs in 40-70% of children with sarcoidosis [2]. In addition, many children with sarcoidosis have cutaneous manifestations, such as erythema nodosum, papules, plaques, nodules, and changes in pigmentation [1]. Erythema nodosum can be seen in 31% of children with sarcoidosis [2]. Many children with sarcoidosis also have ocular involvement. Most commonly, patients have uveitis, but choroidal or conjunctival granulomata can also be seen [2]. Other organ systems, including the liver, can also be involved [1]. While genetic mutations in CARD15/NOD2 can be demonstrated in early onset sarcoidosis and Blau syndrome, there has not been a definitive genetic mutation associated with adult sarcoidosis or sarcoidosis in older children, and mutations in CARD15/NOD2 are not found in these patients [2].

Alagille syndrome is a heritable form of chronic liver disease in childhood associated with a mutation in *JAG1*. Patients with this syndrome have hypoplastic intrahepatic bile ducts with patent extrahepatic bile ducts [7-9] Other findings of the syndrome can include congenital heart disease, characteristic facial features, butterfly vertebrae, growth retardation, renal abnormalities, abnormal eye findings, pancreatic disease, and intracranial hemorrhage [7-9]. The most common form of cardiovascular involvement in Alagille syndrome is peripheral pulmonary artery stenosis. Liver biopsy reveals intrahepatic cholestasis in association with a relative paucity of intrahepatic interlobular bile ducts [7-9]. A prior association of Alagille syndrome and sarcoidosis has not been previously reported.

## Case presentation

A 2-year-old male with a known history of genetically confirmed Alagille syndrome presented to the hospital with tachypnea, respiratory distress, and a fever of 104°F. He had multiple hospitalizations in the preceding 3 months for similar symptoms and had been diagnosed with pneumonia 3 times and cholangitis once. He had recently finished a course of intravenous antibiotics for presumed pneumonia 2 days prior to presentation.

**Figure 1:**
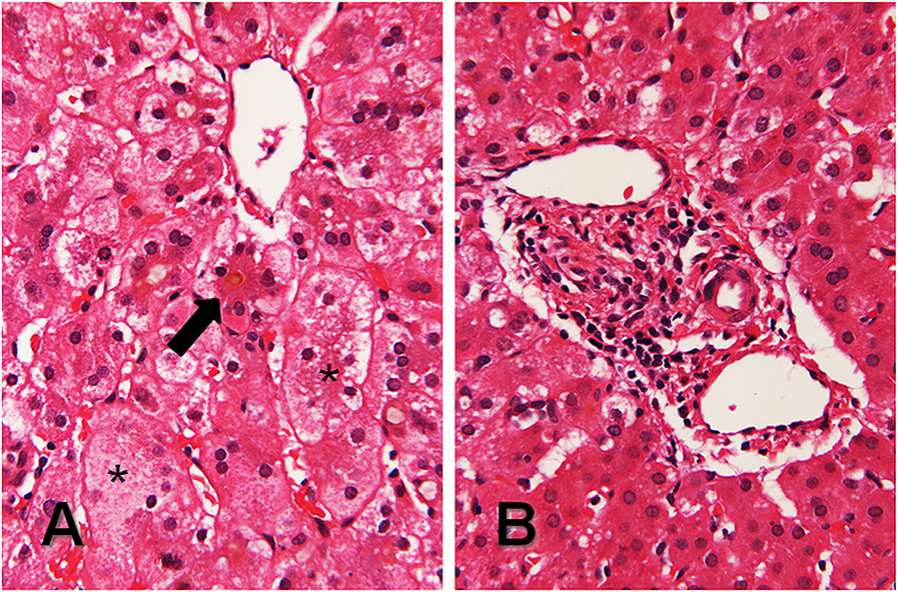
**Liver biopsy at nine weeks consistent with Alagille syndrome. **(**A**) Canalicular cholestasis (arrow) with scattered hepatic giant cells containing eight to ten nuclei (asterisks), and (**B**) a representative portal tract containing two small dilated veins, a small artery, and no recognizable bile ducts. (**A** and **B**, hematoxylin and eosin, 132x original magnification).

**Figure 2:**
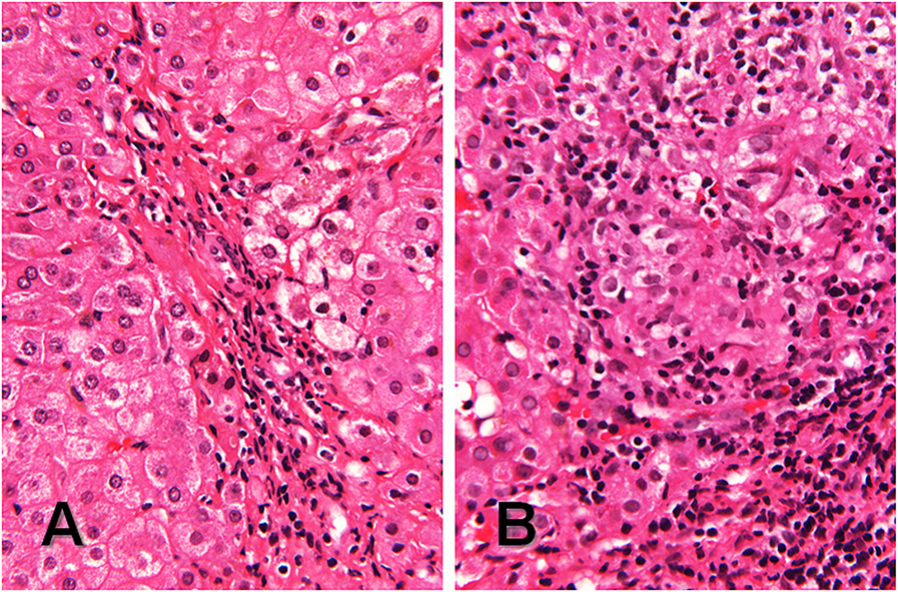
**Liver biopsy at 26 months consistent with both Alagille syndrome and sarcoidosis.**(**A**) Small portal tract with inconspicuous blood vessels and no recognizable bile ducts, no cholestasis, and no giant cell transformation in the hepatic parenchyma, and (**B**) a granuloma with a central cluster of epithelioid histiocytes partially surrounded by a band of small mature lymphocytes. (**A** and **B**, hematoxylin and eosin, 132x original magnification).

The patient’s past medical history was significant for Alagille syndrome which presented as direct hyperbilirubinemia at 2 months of age. Liver biopsy was performed at 3 months and revealed cholestasis, paucity of bile ducts and giant cell transformation (Figure 1), consistent with Alagille syndrome. He was also noted by imaging and clinical examination to have butterfly vertebrae, right and left pulmonary artery stenosis, and prominent forehead and eyes. The diagnosis was later confirmed by gene sequencing revealing that the patient had a novel G to A point mutation in exon 20 of *JAG1,* which changes a tryptophan to a premature stop codon, a genetic result that would be consistent with the clinical diagnosis of Alagille syndrome. In addition, the child had severe failure to thrive. The child had a repeat liver biopsy (Figure 2) two months prior to presentation that revealed, in addition to signs of chronic liver disease with paucity of bile ducts and bridging fibrosis, several epithelioid cell granulomata (not typically a feature of Alagille syndrome).

On physical examination, the child had a temperature of 103°F, a heart rate of 156 beats per minute, a blood pressure of 121/53 mm Hg, and had an oxygen requirement of 5 L/minute by face mask. He was tachypneic and grunting. He had shotty lymphadenopathy in his anterior and posterior cervical chains as well as in his inguinal region. His breath sounds were clear and equal bilaterally. He had a 3/6 systolic murmur heard best over the left upper sternal border. He had 2+ femoral and distal pulses, with <2 second capillary refill distally. The child’s abdomen was soft and non-tender, with hepatomegaly, which was baseline for the patient. His spleen tip was not palpable. On the soles of the child’s feet were multiple erythematous palpable nodules.

Initial laboratory studies revealed a white blood cell count of 19.6 thousand cells/μL, with 77% segmented neutrophils, 7% bands, 14% lymphocytes, and 2% monocytes. His hemoglobin level was 10 g/dL, and his platelets were 635,000/μL. His electrolytes were all within normal limits, and his total serum protein was elevated at 8.9 g/dL, with an albumin of 4.2 g/dL.

**Figure 3:**
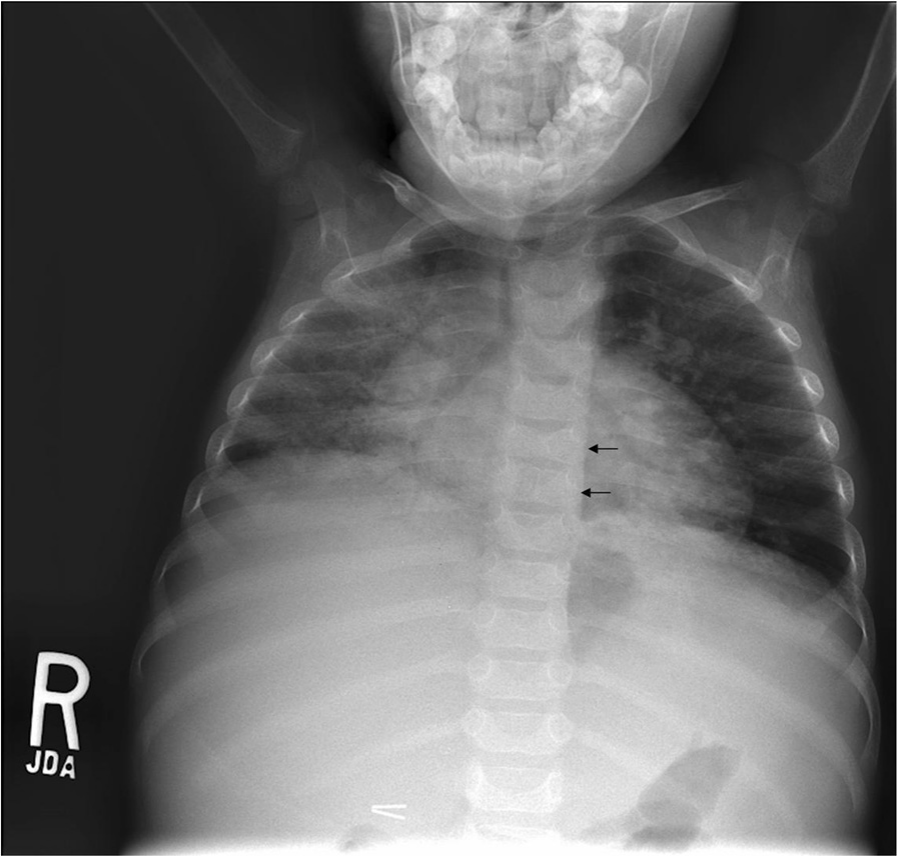
**Chest radiograph consistent with Alagille syndrome and sarcoidosis. **Anterior view chest radiograph revealing extensive bilateral chronic lung disease, mediastinal adenopathy, butterfly vertebrae (arrows), and hepatomegaly.

The child was admitted to the pediatric intensive care unit for one day because of significant respiratory distress and started on intravenous broad spectrum antibiotics. The patient had an echocardiogram that was normal and revealed no vegetations. He had chest, abdominal, and pelvic computed tomography and radiography that failed to reveal an abscess or other obvious infectious process, but did show airspace and interstitial lung disease more on the right than left, including small areas with ground glass appearance, mediastinal and peritracheal adenopathy, butterfly vertebrae, and hepatomegaly (Figure 3). Abdominal ultrasound showed findings consistent with hepatic granulomata. All blood cultures were negative, and a bronchoalveolar lavage was performed that had no hemosiderin-laden macrophages and was negative for bacteria, fungi, pneumocystis, and mycobacteria. The patient had a negative tuberculosis skin test and 3 morning gastric aspirates that did not reveal mycobacteria.

**Figure 4:**
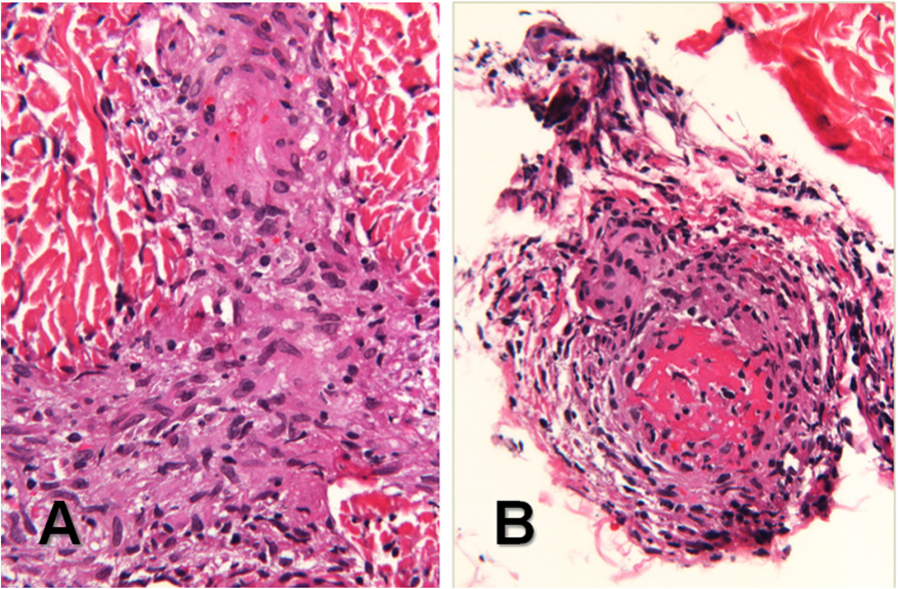
**Skin biopsy from the right foot at 28 months. **(**A**) Granulomatous angiitis with obliteration of the vascular lumina and slight red cell extravasation in the dermis, and (**B**) a thrombosed blood vessel with a granulomatous reaction at the dermal-subcutis junction. (**A** and **B**, hematoxylin and eosin, 132x original magnification).

Rheumatology was consulted when the infectious evaluation had failed to produce a diagnosis and his C-reactive protein and erythrocyte sedimentation rate (ESR) were elevated at 1.69 mg/dL and 80 mm/hr, respectively. An ophthalmologic examination revealed diffuse creamy white infiltrates and vascular sheathing consistent with vasculitis in both eyes. A skin biopsy of a nodule on the plantar aspect of his foot showed a granulomatous vascular reaction, consistent with granulomatous angiitis (Figure 4). A serum ACE level was 76 U/L (reference range: 13–100). The child’s serum lysozyme level was elevated at 11.9 μg/mL (reference range: 4–10.3). There was concern that the child may have been having some joint pains in his knees at home. Also, during his hospitalization, he indicated pain in his left elbow and had some mild joint swelling. Based on the constellation of findings and the granulomata on the liver biopsy performed 2 months prior, Rheumatology was concerned for sarcoidosis and recommended that the patient be treated with high dose glucocorticoids for 3 days (30 mg/kg/day of intravenous methylprednisolone). After the first dose, the child’s tachypnea began to improve, his oxygen requirement resolved, and he became more active. Following the third dose, the child was started on 2 mg/kg/day of oral prednisolone. Mycophenolate mofetil 200 mg twice a day was also initiated rather than methotrexate because of concern of additional liver toxicity in a child with Alagille syndrome, and the patient received an infusion of infliximab of 10 mg/kg. The child’s fevers, joint pains, tachypnea, oxygen requirement, and rash had resolved 4 days after starting pulse corticosteroids.

The child was discharged home on prednisolone (2 mg/kg/day) and mycophenolate mofetil with monthly infliximab infusions. One month following discharge, his ophthalmologic examination had returned to normal. The child initially did well, and a steroid dose wean and decrease in mycophenolate mofetil to 200 mg once daily were initiated. Shortly after this change in medication dosages, the child began having fatigue, joint swelling, and an elevation in ESR. The child’s infliximab dose was increased to 20 mg/kg and the dosing interval was shortened to 3 weeks. His prednisolone dose was increased until his next infliximab infusion. The child continued to not tolerate steroid weaning, so his infiximab infusion interval was decreased to 2 weeks and mycophenolate was returned to twice daily dosing to help decrease the steroid burden. The child has subsequently tolerated a steroid wean down to 0.2 mg/kg/day of prednisolone. His infliximab and methylprednisolone infusions have been spaced to every 3 weeks, and his arthritis, uveitis, and pleuritis have been under good control.

## Discussion

The coexistence of Alagille syndrome and granulomatous disease has not been previously described in the literature. This case report highlights the potential for concurrent rare conditions in a single pediatric patient. Alternatively, these conditions may be related in an unrecognized way.

The diagnosis of inflammatory granulomatous disease in this patient was complicated by several factors. The patient’s diagnosis of Alagille syndrome could explain some of his clinical features, such as hepatomegaly and failure to thrive [7]. In addition, there was a reluctance to consider two rare diagnoses in one patient [10]. While this 2-year-old child did have rash, arthritis, and eye findings, his pulmonary involvement, lymphadenopathy, and constitutional symptoms of fever and poor growth represent a presentation rarely seen in early-onset sarcoidosis [1]. The skin rash in this child clinically resembled erythema nodosum; however, morphologically, it consisted of granulomatous angiitis (with microthrombi) and primarily involved the dermis with no evidence of septal panniculitis. This cutaneous vascular manifestation of sarcoidosis is extremely rare in adults and not previously reported in children [11-14]. Vascular abnormalities, such as vessel wall abnormalities, aneurysms, vascular stenoses, moya moya, renovascular hypertension, and intracranial hemorrhage [15,16] can be seen in Alagille syndrome, though diffuse granulomata consistent with sarcoidosis have not been described. A young girl with Takayasu arteritis and Alagille syndrome [15] represents the only known reported case of Alagille syndrome associated vasculitis.

Sarcoidosis patients may have hypercalcemia secondary to extrarenal production of calcitriol leading to increased intestinal absorption of calcium [17]. This child was being treated for hypocalcemia secondary to gastrointestinal losses due to his Alagille syndrome. Thus, it is possible that the child’s Alagille syndrome masked a tendency toward hypercalcemia.

This child’s atypical presentation of sarcoidosis and the rarity of his two conditions suggest a possible link between them. Although Alagille syndrome is not typically associated with granuloma formation, in one study, there was a patient with Alagille syndrome who developed a cystic mandibular mass, which on biopsy revealed multiple central giant cell granulomata that were nonmalignant [9]. This case causes speculation that perhaps granulomatous disease could be a manifestation of Alagille syndrome. Our patient had granulomata on a liver biopsy during a previous evaluation 2 months prior to presentation, which at the time of examination, had been attributed to his Alagille syndrome. Recent reviews of hepatic granulomas report an incidence of approximately 4% in unselected liver biopsies in adults. The causation of hepatic granuloma can be determined in most cases and varies by region; noninfectious immune categories predominate in Western countries, where infectious causes predominate in the developing world [18]. Nevertheless, for this child, the constellation of uveitis, arthritis, pleuritis, and rash in the setting of an elevated ACE level and granulomas on biopsy strongly argue in favor of coincident diagnosis of sarcoidosis.

There may be a molecular basis for our patient’s constellation of findings. The two genes associated with Alagille syndrome are *JAG1* and *NOTCH2*[16]. *JAG1* encodes a cell surface ligand for one of the Notch transmembrane receptors. The role of the Notch signaling proteins is not well understood, but is highly conserved; *JAG1* is on chromosome 20 and accounts for 70% of the gene mutations in Alagille syndrome [7]. There are four mammalian Notch receptors (1–4) and five Notch ligands (Jagged1, Jagged2, Delta-like 1, Delta-like 3, and Delta-like 4) [19]. Of note, *JAG1* mutations are not specific for Alagille syndrome [16]; in a large kindred study, *JAG1* mutations were associated with congenital right heart obstructive disease without other diagnostic criteria of Alagille syndrome [20].

It is known that the Notch signaling pathway is important for regulation of angiogenesis [21]. Notch-1 has also been recognized as a T cell oncogene and is highly expressed in the thymus. Specifically, immature lymphoid cells that enter the thymus differentiate along the B cell lineage without Notch signals [19,22]. Notch-1 may have implications for further T cell lineage differentiation. In one mouse model, thymocytes with a functional γδ T cell receptor (TCR) were diverted to the αβ lineage with a constitutive Notch-1 signal [22]. In addition to TCR lineage, Notch proteins also seem to regulate CD4 T_H_ cell differentiation; however, there are reports of Notch signaling promoting both T_H_-1 and T_H_-2 cell polarization [19]. CD4 T cells incubated with Jagged1-expressing antigen presenting cells (APC) were induced to a T_H_-2 cell fate, and those incubated with APC that over-expressed Jagged1 were induced to a regulatory T cell fate [19]. Thus, *JAG1* mutations, like those seen in Alagille syndrome, are likely to alter T cell development within the thymus and modify effector CD4 T cell differentiation in the peripheral lymphoid organs. Taken together with the evidence that over-expression of Jagged1 on APC promotes T_reg_ and T_H_-2 CD4 T cell development [19] and intact Notch signaling is required for appropriate populations of TCRγδ T cells and TCRαβ T cells [19,22], then it follows that Jagged1/Notch interactions are critical to immune homeostasis.

The genetics of sarcoidosis are being investigated. There are candidate genes located on chromosomes 5 and 6. One such candidate gene is BTNL2 in the major histocompatibility complex II region on chromosome 6 [2]. Blau syndrome has been associated with mutations in CARD15 /NOD2 which results in a gain of function mutation; similar mutations have been demonstrated in cases of early onset sarcoidosis [5,6]. The CARD15/NOD2 mutations are associated with increased basal NF-κB (nuclear factor κB) activity independent of muramyl dipeptide, a component of bacterial peptidoglycan, and a ligand for CARD15 in phagocytic cells [5]. The NF-κB signaling pathway is preferentially required for the production of interferon γ (IFNγ) through a CD4 T_H_-1 cell mediated pathway [23].

Granulomas are formed by activation of T_H_-1 cells and macrophages; thus, sarcoidosis is often considered a T_H_-1 mediated disease. Granulomas in early sarcoidosis express a strong T_H_-1 phenotype with production of interleukin-2 and IFNγ, which results in a positive feedback loop to recruit additional T_H_-1 cells and IFNγ production [18,24]. Therefore, if CARD15/NOD2 mutations result in increased basal NF-κB activity, which predispose to a T_H_-1 cell preference and production of IFNγ, the resultant phenotypes would have a diffuse granulomatous process [5,18,23,24].

The child described herein has a mutation in *JAG1* which results in a truncated protein and is predicted to result in abnormal or no Jagged1/Notch binding. Without this signaling, a predominance of T_H_-1 phenotype and uncontrolled TCRγδ T cells would be expected, which would be similar to the immune phenotype seen in Blau syndrome with a resultant diffuse granulomatous process [5].

## Conclusion

In conclusion, this child has a previously unreported combination of Alagille syndrome and inflammatory multi-system granulomata consistent with sarcoidosis. In patients with unusual symptoms, consideration of rare conditions is warranted even when they have other known and seemingly unrelated rare conditions. Although this is the first published report of sarcoidosis and Alagille syndrome in the same child, a *JAG1* mutation would seem to predispose a patient to immunologic dysregulation and the development of granulomas.

## Consent

Written informed consent was obtained from the child’s mother for publication of this case report and any accompanying images. A copy of the written consent is available for review by the Editor-in-Chief of this journal.

## Abbreviations

ACE: Angiotensin converting enzyme; CARD15: Caspase recruitment domain 15; NOD2: Nucleotide binding oligomerization domain 2; ESR: Erythrocyte sedimentation rate; TCR: T cell receptor; APC: Antigen presenting cell; NF-κB: Nuclear factor κB; IFNγ: Interferon γ.

## Competing interests

The authors declare that they have no competing interests.

## Authors’ contributions

MM and MZ drafted the manuscript. TB and RQC were involved in the patient care, participated in the manuscript's design and coordination, and critically revised the manuscript. DK provided figures and assistance with histological data and assisted with manuscript revision. All authors have reviewed the final manuscript and gave approval for publication.
